# Supplementary material for: Are carbon emissions trading and green financial instruments synergistic? -Comprehensive quantitative research based on content analysis
Source: PLoS One. 2024 Mar 7;19(3):e0298601. doi: 10.1371/journal.pone.0298601 (PMC10919601; doi:10.1371/journal.pone.0298601)
Supplement: S1 Data — (ZIP) [file pone.0298601.s001.zip › Data/地级市Z_F工作报告.zip!/░▓╗╒/╗╞╔╜╩╨/└┤╫╘╣1⁄2╓┌║┼í╛┬φ┐╦╩2╛▌═°í┐_2014.docx]

各位代表：

　　现在，我代表市人民政府，向大会作工作报告，请予审议，并请市政协委员和其他列席人员提出意见。

**一、2013年工作回顾**

　　刚刚过去的2013年，面对经济持续下行压力，面对多重罕见自然灾害，全市人民在省委、省政府和市委的坚强领导下，坚持以党的十八大和十八届二中、三中全会精神为指导，紧紧围绕建设现代国际旅游城市战略目标，牢牢把握稳中求进工作总基调，统筹稳增长、调结构、促改革、惠民生，克难而进，扎实工作，夺取了防汛抗洪和抗旱救灾全面胜利，较好完成了市六届人大一次会议确定的工作任务。

**一是经济保持平稳增长。**初步核算，全市实现生产总值468亿元，同比增长9%；财政收入81.02亿元，增长5.4%；全社会固定资产投资526.7亿元，增长18.3%；社会消费品零售总额196.2亿元，增长14%；外贸进出口总额8.03亿美元，增长14.6%；市内金融机构贷款余额472亿元，增长13.2%。工作中，我们认真落实省、市促进经济持续健康较快发展和大力发展民营经济各项政策措施，积极化解经济运行不利因素，促进三次产业协调发展。农业增加值53亿元、增长3%，规模以上农产品加工企业205家，实现产值173.7亿元、增长16.9%；茶叶产量3.1万吨、增长4.7%，出口1.02亿美元、增长43.8%。净增规上工业企业48家，规上工业增加值118.8亿元、增长14.2%；高新技术产业增加值占规上工业增加值比重达38.8%，新增国家高新技术企业9家。市和歙县经济开发区被列入全省产城一体化试点，全市园区完成固定资产投资150亿元，实现工业总产值460亿元。持续加大旅游营销力度，成功举办第十七届中国黄山国际旅游节暨徽文化节等节庆赛事活动，全年接待游客3732.6万人次，旅游总收入314.5亿元，分别增长2.5%和3.8%。服务业增加值198亿元，增长8%。黄山旅游股份公司获得省政府质量奖、首届中国质量奖提名奖。

**二是重点工作扎实推进。**全力扩大有效投入，“十大工程”完成投资521亿元、增长12.8%，黄山北站、歙县北站、歙黟一级公路、宁国至黄山天然气支线工程等重点项目开工建设，京福高铁加快建设，黄祁高速建成通车，西海大峡谷观光缆车等140个重点项目竣工运营。加大招商引资力度，全年到位内资564.5亿元、增长20%，利用外商直接投资2.53亿美元、增长15.4%。国家服务业综合改革试点中期评估通过省复核,黄山现代服务业产业园获省政府批准设立。黄山风景区荣获全球首家优秀目的地旅游景区，古徽州文化旅游区5A景区创建通过国家旅游局景观质量等级评定。实施新安江流域综合治理项目100个,出境断面水质总体保持优良，太平湖入选全国15个江河湖泊生态环境保护项目。节能减排完成省下达目标任务。“百村千幢”古民居保护利用工程目标任务完成。全市百佳摄影点增至166个。扎实开展关键区域控制和重点村镇环境风貌整治，完成改徽8561幢。强力推进千万亩森林增长工程和绿色质量提升行动，全面启动政策性森林保险，造林绿化16.57万亩。休宁县入选全国生态文明先进县，全市新增国家级生态乡镇6个，再次荣获“中国最佳休闲城市”称号。

**三是城乡建设步伐加快。**市经济开发区和高铁新区成功整合，高铁站场等重点项目加快推进，新区快速通道启动建设。徽州文化与生态长廊城市展示馆、文化广场主体工程完成，文峰桥建成通车，江心洲公园、戴震公园改造和屯光大道、齐云大道绿化提升全面完成，市图书馆、档案馆、残疾人综合服务中心等公益性项目全面建成，新增和优化公交线路8条，更新公交车40辆。加快推进区县城和建制镇建设，屯溪区黎阳印象、黄山区北大门沿线综合改造提升、徽州区颖溪河两岸及岩寺老街整治、歙县客运站等一批重点项目全面完成，休宁县旧城改造、黟县新城区路网和祁门县新城区开发等城镇建设项目快速推进，全市城镇化率达48.8%，提高1.7个百分点。扎实开展美好乡村建设，全市村庄布点规划和第一批106个中心村建设规划编制完成，新建、续建项目1498个， 36个省级重点示范村完成建设任务。西递村荣获“中国十大最美乡村”称号，庄里等9个村跻身全国“美丽乡村”创建试点。

**四是民计民生持续改善。**深入开展“七保”工作，精心组织实施37项省市民生工程，完成投资31.98亿元，惠及148万城乡居民。实施更加积极的就业政策，城镇新增就业2.7万人，下岗失业人员再就业1.7万人，城镇登记失业率控制在4.2%以内。着力扩大社会保障覆盖面，五项社会保险参保93.9万人次，城乡居民社会养老保险参保79.4万人。提高企业离退休人员养老金、城乡低保、重点优抚和五保对象补助标准，全面实现市内异地就医即时结算，未参保集体企业退休人员基本生活得到保障，被征地农民社会保障制度全面实施。新农合参合率101.2%，大病保险全面启动，城乡医疗救助16.46万人次，发放救助资金2676.48万元。开工建设各类保障性住房和棚户区改造住房7952套，基本建成13266套，超额完成年度任务。完成农村危房改造1.66万户，8个灾后倒房重建集中安置点基本建成，因灾倒房的群众春节前将全部入住新居。全力推进扶贫攻坚，实施扶贫项目568个，投入资金4.8亿元，2.5万人实现稳定脱贫。全市城镇居民人均可支配收入23230元，农民人均纯收入10550元，分别增长9.5%、15%，增速均高于全省平均水平。

**五是社会事业全面发展。**新增院士工作站2家、省级工程技术研究中心3家，黄山茶产业技术研究院挂牌成立，我市蝉联全国科技进步先进市。新建、改扩建和增设附属幼儿园85所，完成35所义务教育阶段学校标准化建设，黄山茶业学校整建制并入黄山职业技术学院。新增中国传统村落27个、国家级重点文物保护单位15处。市人民医院、市中医院、市第二人民医院实施改扩建，黄山首康医院建成运营，县级公立医院改革初见成效。人口自然增长率控制在3.5‰以内，继续保持低生育水平。全民健身运动蓬勃开展，成功举办市第六届运动会暨第二届残疾人运动会，在十二届全国运动会实现金牌零的突破。深入开展安全生产大检查，12项安全生产指标控制在省下达范围之内。夯实社会治理基础，建成32个精品社区。扎实开展“六五”普法，区县社区矫正中心全面建成投入使用。加大矛盾纠纷排查化解力度，畅通信访渠道，全市信访形势总体平稳。加强社会治安防控体系建设，严厉打击各类违法犯罪行为，荣膺全国社会管理综合治理优秀市“三连冠”，胜利捧回“长安杯”。国防教育、国防动员、民兵预备役、人民防空和双拥优抚安置工作不断加强，荣获“全省双拥模范城”六连冠。妇女、未成年人、老年人、残疾人权益得到保障。哲学社会科学、文学艺术、新闻出版、地方志、档案工作得到加强。气象、地震、防灾减灾工作取得新进展，民族宗教、外事侨务、对台等工作取得新成绩。

**六是自身建设不断加强。**严格执行中央“八项规定”和省、市委有关规定，切实加强政府自身建设，努力提升推动发展、服务群众的能力和水平。坚持科学民主决策，大力推进依法行政，自觉接受市人大及其常委会的法律监督、工作监督和市政协的民主监督，市政府系统承办的452件人大代表建议和政协提案全部办结。修订完善市政府工作规则，进一步加强政府投资项目、政府性资金、政府性债务和政府采购、工程招投标、土地出让管理等制度建设。深化行政审批制度改革，清理行政审批项目360项，取消39项、下放27项、冻结20项，市政务服务中心全年受理各类行政审批及服务事项16.25万件，按时办结率达99.24％。坚持勤俭办一切事业，“三公”经费支出同比下降15.84%。认真落实党风廉政建设责任制和《廉政准则》，扎实开展廉政风险防控，强化行政监察和审计监督，严肃查处了一批违纪违法案件。

　　各位代表，过去的一年，我们能够战胜困难和挑战，取得来之不易的成绩，是省委、省政府和市委坚强领导的结果，是市人大及其常委会和市政协监督支持的结果，是全市人民凝心聚力、真抓实干的结果。在此，我代表市人民政府，向全市广大工人、农民、知识分子、干部，向驻黄解放军指战员、武警官兵和政法干警，向各民主党派、各人民团体和各界人士，致以崇高的敬意！向关心、支持黄山现代化建设的中央、省驻黄单位和海内外友好人士，向在我市创业的投资者、建设者，表示衷心的感谢！

　　在肯定成绩的同时，必须清醒认识到经济社会发展中存在的困难和问题。主要是：经济持续增长的基础不够稳固，发展不足、发展不优的问题比较突出，财政收支矛盾压力加大，部分指标未实现预期目标；产业支撑力不强，农业产业化水平不高，工业缺乏有竞争力的产业和有带动力的龙头企业，服务业综合效益有待提升;中心城区人气不足，首位度不高，带动力不强；部分城市低收入群体和深山区、库区、革命老区群众生活依然困难；教育、医疗、就业和社会保障等方面还存在一些薄弱环节；节能减排、安全生产、社会治理等任务仍然十分繁重；少数政府部门和工作人员发展意识不强、工作谋划不深、落实推动不力，形式主义、官僚主义、享乐主义和奢靡之风还不同程度存在。对此，我们一定要高度重视，采取切实有效措施，认真加以解决，不辜负全市人民和各位代表的期望和重托。

**二、2014年主要工作**

　　2014年是全面贯彻落实党的十八届三中全会精神、全面深化改革的开局之年，也是实现“十二五”规划目标任务的关键一年。今年政府工作的总体要求是：全面贯彻落实党的十八大、十八届二中、三中全会、中央经济工作会议和省委九届九次全会、省经济工作暨城镇化工作会议精神，按照市委五届七次全会的总体部署，坚持稳中求进工作总基调，以全面深化改革为根本动力，扎实开展“提质增效、转型升级推进年”活动，着力加强生态环境保护和文化传承，着力激发市场活力，着力增加有效投入，着力优化产业结构，着力促进城乡统筹，着力保障和改善民生，促进经济持续健康较快发展和社会和谐稳定，在科学发展、富民强市征程上迈出更加坚实的步伐。

　　今年经济社会发展的主要预期目标是：全市生产总值增长9.5%；财政收入增长11%；全社会固定资产投资增长15%；社会消费品零售总额增长12%；城镇居民人均可支配收入增长10%，农民人均纯收入增长11%；居民消费价格涨幅控制在3%左右；城镇登记失业率控制在4.2%以内；万元生产总值能耗、主要污染物排放总量控制在省政府下达目标以内。重点抓好以下十个方面工作：

**（一）强化项目带动，持续扩大有效投入**

　　项目建设是提质增效、转型升级的基础。按照经济工作项目化、项目工作责任化的要求，突出抓好重点项目建设，着力增强发展后劲。

　　加快推进项目建设。全力服务好国家和省重点项目建设，积极稳妥推进政府投资类项目建设，大力支持社会投资类项目建设。重点抓好已申报的217个省“861”重点项目建设，确保全年开竣工亿元以上项目各100个以上。基础设施方面，加快推进京福高铁、歙黟一级公路、宁国至黄山天然气支线工程建设；开工建设黄杭铁路，确保高铁客运枢纽、玉屏索道改造主体工程等项目基本建成；完成月潭水库项目可研报告和相关专项报告编制；扎实做好皖赣铁路扩能、济祁高速、芜黄高速、黄千高速、黄山机场4E改造等重大项目前期工作。产业发展方面，加快推进永新股份绿色软包装、东晶光电、紫光优蓝机器人、金普森太阳能光热综合利用、甲骨文黄山基地和卢村悦榕庄等项目建设；开工建设福派园食品产业园、智谷科技园、中国物联网黄山研发基地、河马文化产业园、百乐门国际文化娱乐经贸广场等项目；力争蓝猫动漫、康师傅瓶装水、祥源红茶等项目建成投产，希尔顿酒店、喜来登酒店、太平湖皇冠假日酒店建成运营。

　　提升项目工作水平。切实抓好项目谋划工作，认真研究国家和省产业导向，有针对性地谋划编制一批有利当前、事关长远的产业发展类和基础设施类重大项目，确保项目对上能争取、对外能招商。切实加强项目管理，强化部门联动，规范审批流程，扎实做好项目报批各项前期工作，力争有更多的项目列入国家和省重大发展规划及省“861”计划，在用地指标和金融信贷等要素保障上获得更多更大支持。切实加大项目推进力度，坚持以产业项目为重点，统筹推进民生工程、基础设施和生态文化保护等各类项目建设，多上有利于产业优化升级的项目，扩大能够提供实实在在就业和税收的投入，特别是要善于调动市场力量参与项目建设及后期运营。进一步落实项目工作责任制，着力提高效率、简化程序、优化服务，让签约项目尽快落地开工、建成投产，转化为现实生产力。

**（二）推动转型升级，着力增强产业支撑力**

　　产业发展是提质增效、转型升级的重点。按照做精一产、做强二产、做优三产的思路，全力发展实体经济，大力培育龙头企业，不断提升产业素质。

　　做大做强工业经济。以新型工业化发展为导向，坚持存量调整和增量优化并举，突出抓好190个、总投资117.7亿元的工业转型升级项目建设。加快发展电子信息、机械制造、新型材料、绿色食品等优势产业，改造提升精细化工、纺织服装等传统产业，培育引进智能制造、信息软件、生物医药、新能源等战略性新兴产业，加快上下游企业配套，力争七大优势产业规上企业数、工业产值均占全市80%以上。加快企业科技、管理、品牌和营销模式创新，加大30户重点骨干企业扶持力度，支持现有489户规上工业企业扩张规模，全年新增规上企业50户，规上工业增加值增长14%以上；加快推动小微企业向“专、精、特、新”方向发展，新认定国家高新技术企业、创新型试点企业11家以上。推动园区由粗放低效型向集约高效型发展，引导各园区重点培育1～3个主导产业，主导产业占园区比重提高2个百分点以上。对园区低效企业、低效用地进行全面清理，加快腾笼换鸟步伐，力争全市开发区投资强度和亩均税收提高20%以上。

　　大力发展现代农业。坚持特色化布局、标准化生产、产业化经营，大力发展精致农业，力争农业增加值增长3.5%以上。以提升产业集中度为重点，通过改良茶苗、发展规模种植、引进外部资源并购、入股现有加工企业，着力提升茶产业市场竞争力，全年新发展无性系良种茶园2万亩，茶叶总产3.2万吨。加快发展毛竹、油茶、菊花、蚕桑、中药材等特色种植，做好食用菌、果蔬和养殖等“土、特、绿”文章，发展各类特色基地7万亩，新增省级“一村一品”示范村镇3～5个。大力发展林下经济，争创省级林下经济示范点20个。高起点、高标准抓好3个省级、10个市级现代农业园区建设，积极争创国家级现代农业园区。大力发展农产品精深加工业，新增农民专业合作社100家、规模以上农产品加工企业10家。加强良种良法配套、质量安全、全程机械化等农业实用技术推广。继续办好黄山茶叶暨名优农产品推介活动，打响绿色生态品牌。

　　加快旅游转型升级。以文化旅游生态融合发展为主攻方向，高水平推进皖南国际文化旅游示范区建设。加快引进培育休闲度假、摄影写生、文化产品展示交易、户外运动、养生养老等体验性、参与性强的新型业态，促进全市旅游由观光型向复合型转变。加快黄山风景区国际精品旅游景区建设步伐，确保古徽州文化旅游区通过国家5A景区评审。大力发展乡村旅游，规范发展一批有特色、有品位的乡村客栈。制定实施中长期营销规划，推进旅游目的地整体营销，集中力量，持续投入，打造“大黄山”旅游品牌。发挥政策杠杆作用，调动旅行社等旅游企业积极性，加大客运沿线、通航城市营销力度，开发东南亚、俄罗斯等境外市场，提高旅游营销的针对性和实效性。深入开展国家智慧旅游城市试点，加大新媒体营销力度。强化旅游市场监管和服务,力争旅游接待量增长8%，旅游总收入增长10%，入境游客增长10%。

　　积极发展现代服务业。深入开展国家服务业综合改革试点，实施服务业重大项目160个以上，力争服务业增加值增长10%以上。拓展和完善服务业体系，大力发展现代物流、文化创意、研发设计、商务会展等生产性服务业，积极发展文化旅游、康体养老、家政服务等生活性服务业。推进黄山现代服务业产业园建设，抓好三个省级服务业集聚区建设，启动徽州古城、齐云山等服务业集聚区建设。开工建设苏宁广场、黄山颐高广场，引进1～2个大型物流项目，建成市农产品批发市场。引导合理的房地产消费，保持房地产市场健康发展。

**（三）推进开放发展，大力推动招大引强**

　　招商引资是提质增效、转型升级的重要抓手。必须以更大的气魄实施对外开放，把一流的资源配置给一流的企业，以共赢发展的理念推进招大引强，加快培育竞争新优势，提升发展新水平。

　　突出抓好招商引资。以国内外500强、中央和省属大企业、知名民企为主攻方向，以长三角、珠三角等沿海地区和港澳台地区为重点区域，围绕发展工业优势产业、现代服务业和现代农业，积极承接产业转移，大力开展招商引资。力促国药祁红、开元农机产业园、黄山风景区国家地理3D影院、冠军疗养基地等签约项目开工建设，推进娃哈哈山泉水、口子酒业、华侨城、万达、红星美凯龙、青岛伟业和智慧黄山等在谈项目签约落地，再精选并跟踪一批新的企业和项目。进一步完善招商工作机制，大力推行产业链招商、专业团队招商，注重发挥商会、协会作用推进以商引商，全年新签亿元以上项目超过100个，到位内资增长12%，外商直接投资增长10%。

　　着力扩大对外开放。进一步扩大国际交流合作，加强与友好城市、友好景区的交往，做好外事侨务和台湾事务工作。力争台湾居民口岸落地签注获批，新开1～2条国际包机航线。认真办好第十八届中国黄山国际旅游节暨徽文化节、山地车节等活动。加大中小外贸企业扶持力度，加强茶叶、精细化工、高新电子等特色优势产业出口基地建设，促进对外贸易稳定增长。鼓励企业引进国外关键设备和先进技术。全年新增1000万美元以上出口企业10家，外贸进出口总额增长10%。

**（四）统筹城乡发展，抓好城镇和美好乡村建设**

　　加快推进以人为核心的新型城镇化，促进城乡公共服务均等化，努力形成城乡各具特色、共同繁荣发展的生动局面，城镇化率达50%。

　　加强城镇规划管理。坚持以强化产业支撑保障就业、以完善公共服务保障安居、以有序推进农业转移人口市民化保障城镇化健康发展的工作思路，推动经济社会发展、城乡建设、土地利用、产业布局、生态环境保护规划等“多规合一”。认真抓好新一轮市域城镇体系规划和修编，加快中心城区规划编制，优化区县城总体规划，完成全市95个乡镇规划编制。切实加强重点风景区、关键区域规划管理，真正做到不规划不开发、先布局再落子，永葆青山绿水的鲜明特色。

　　提高城镇建设水平。市中心城区计划安排政府投资项目69个、社会投资项目68个，完成固定资产投资224亿元，增长25%。坚持产城一体发展，加快推进市经济开发区建设，打通新区快速路，完成新安江上游段综合治理工程和高铁站场项目土建工程，进一步拉开城市框架，促进南部城镇群集聚发展。改造提升新园路、黄山路等城市道路，完成老城区污水管网改造，敷设天然气管网13公里，改造供水管网15公里；启动高铁水厂建设，完成一水厂制水厂迁建、第二污水处理厂建设。坚持统一规划，分步实施，改造老旧小区25个、棚户区5个。加快推进区县城基础设施项目建设，继续抓好47个建制镇建设，支持屯光等8个镇争创全国重点镇，区县城和建制镇分别完成固定资产投资195亿元、108亿元，分别增长17%和20%。扎实开展国家智慧城市试点，强化交通和市容市貌整治，规范小区物业和各类市场管理，不断提升城镇管理水平，争创“联合国人居奖”城市。

　　推进美好乡村建设。以环境整治为突破口，以强化产业支撑为重点，突出抓好“八项重点任务”，确保首批36个省级重点示范村顺利通过考核验收，全面开展第一批尚余70个中心村建设，统筹推进其他村庄建设。抓好世行贷款新农村建设项目，做好与省属企业的结对共建工作，引导更多社会力量参与美好乡村建设，力争在项目建设、产业发展等方面取得更大成效，美好乡村建设走在全省前列。扎实开展“三线三边”整治，全面完成广告标牌、省市交界点、景区周边等10个方面、1000多个整治点的治理工作，使全市城乡环境有明显改善。突出抓好扶贫攻坚“百村千户双联”工程，提升贫困村和贫困家庭自我发展能力，力争2.2万人稳定脱贫。切实加强乡村基础设施和公共服务设施的运营管护，确保发挥持久效益。

**（五）全面深化改革，切实增强发展活力**

　　按照中央改革总体部署和省、市委实施意见，把全面深化改革贯穿到政府的各项工作中，坚持以问题为导向，突出经济体制改革重点，着力在重要领域和关键环节改革上取得实质进展。

　　进一步突出市场导向。按照“使市场在资源配置中起决定性作用和更好发挥政府作用”的要求，正确处理好政府和市场的关系，加快政府职能转变，该由市场决定的，一律按市场规则办；可以由社会、企业投资的项目，政府坚决不做，进一步明确政府作为的边界。把优化发展环境作为持久竞争力，积极培育市场主体，全力支持民营经济发展，大力发展混合所有制经济，规范发展各类市场中介组织，充分激发各类人才创业激情。深化行政审批制度改革，承接落实好中央和省取消、下放的审批事项，对于市级行政审批事项，进一步予以清理、削减，去除不必要的前置审批条件，不断用政府权力的“减法”换取市场潜力的“加法”。做好政府机构改革准备工作，加快事业单位分类改革，扎实推进海事管理体制和工商、质监、食品药品监管体制改革，不断提高政府服务效能和水平。

　　有序推进其他各项改革。稳妥推进农村综合改革，培育发展新型农业经营主体，逐步形成以家庭承包经营为基础，专业大户、家庭农场、农民合作社、农业产业化龙头企业为骨干，其他组织为补充的新型农业经营体系。深化农村土地制度改革，抓好黟县农村综合改革试点，在108个行政村开展农村土地承包经营权确权登记工作，开展农村土地承包经营权抵押贷款试点，规范土地流转程序，力争全市耕地、园地流转面积18万亩。扎实推进林权流转改革，加大林权抵押贷款和森林保险力度，做大做强江南林业产权交易所，加快打造立足黄山、面向全省的专业、规范、统一的森林资源资产交易平台。继续深化社区管理体制改革。扎实推进中心城区城管重心下移体制改革，加快构建“两级政府、三级管理、四级网络”的城市管理体系。有序推进户籍制度改革，全面推行居住证制度，破除城市内部二元结构。巩固基层医药卫生体制改革成果，深化县级公立医院改革，适时启动市级公立医院改革。

**（六）加强保护建设，进一步改善生态环境**

　　以建设美丽中国先行区为目标，以争创国家生态文明先行示范区为契机，持续加大生态建设和环境保护力度，守护好黄山这一方绿水青山。

　　深入开展流域综合治理。实施新安江流域综合治理项目116个，其中补偿机制试点项目52个、完成投资25亿元。建立健全流域垃圾处理长效机制，加快新安江沿线剩余企业搬迁治理，完成流域40家规模化畜禽养殖场污染整治。认真落实国家《千岛湖及新安江上游流域水资源与生态环境保护综合规划》，总结试点经验，争取建立常态化补偿机制。以实施全国江河湖泊生态环境保护项目为契机，全面加强太平湖生态环境保护，统筹做好生态修复、水源地保护、网箱退湖、渔业转型升级、项目建设等重点工作。推进中小河流治理和水系连通工程试点，完成水土流失治理面积90平方公里。加大地质灾害隐患点排查和治理力度，完成歙县金竹村、休宁县阳台村等重点地质灾害点避让搬迁治理工作。扎实推进绿色质量提升行动。认真组织实施退耕还林、森林抚育、造林补贴试点等林业重点生态工程，完成新造林5.85万亩。突出抓好松材线虫病防控，启动实施森林重点火险区综合治理项目，切实加强森林资源保护管理。加强城镇绿化的规划与建设，深入开展国家森林城市创建活动，确保基础指标全面达标，继续创建省级森林城市、森林城镇和森林村庄。启动“国家生态园林城市”创建。

　　突出抓好资源节约集约利用。严格落实节能减排目标责任制，严控污染物新增量，确保完成省政府下达的目标任务。严把项目环保准入关，严格执行环评制度，确保建设项目“三同时”执行率100%。推进祁门东源钨钼矿有序开发。巩固化工、重金属排放企业整治成果，加快淘汰落后产能。推进环境空气质量新标准监测工作，开展集中式饮用水水源地环境综合整治，确保水质达标率100%。推进土地节约集约利用，加快实施土地开发复垦整理项目，实行新增建设用地指标与盘活用好闲置低效用地挂钩，落实土地使用税差别化征收政策，全面开展“批而未供、供而未用、用而未尽”项目清理处置工作，依法收回闲置土地，提高土地利用科学化、精细化水平。

**（七）着力传承创新，增强文化软实力**

　　大力弘扬徽文化特色优势，充分激发文化创造活力，推动文化繁荣发展。加强徽文化资源保护利用。

　　继续推进徽州文化生态保护区建设。组织实施徽州古建筑保护工程，全面加强“十古”保护，积极探索古民居依法规范流转新路子。力争新增中国历史文化名镇、名村8处，中国传统村落15处。全面开展全国第一次可移动文物普查，加强物质和非物质文化遗产保护、管理和利用，推进新安医学非物质文化遗产申报工作。全年新增省级以上非遗生产性保护示范基地5处，非遗生产展示销售区10处以上。创办徽州书院，搭建徽文化研究、传播和推广平台。充分挖掘徽文化精神价值，积极推进非遗项目产业化发展，让传统文化融入时尚、焕发生机。

　　加快壮大文化产业。继续实施重点项目带动战略，力争文化产业增加值增长20%。加快徽文化产业园建设，支持重点文化企业做大做强，争创5家以上省级文化产业示范园区（基地）。发展壮大新兴文化产业，培育特色文化品牌，促进文化与科技融合，重点推进摄影、演艺、写生、动漫等产业发展，扩大徽韵、宏村·阿菊、花山谜窟“激光秀”等演艺品牌影响力。

　　着力提升公共文化服务水平。完善文化设施配套，加强四馆一站（屋）等公共文化场馆建设。继续实施农村电影放映工程，推动数字电视向乡镇、村发展，广播、电视人口综合覆盖率达到96%和98%。拓展农民文化乐园试点工作，广泛开展群众性文化活动，丰富城乡居民精神文化生活。进一步加强文化市场管理，大力发展哲学社会科学、新闻出版和文学艺术，弘扬主旋律、传播正能量，营造昂扬向上、和谐奋进的浓厚氛围。

**（八）抓好财政金融，不断提高支持保障能力**

　　进一步优化财政收支结构，强化金融支持保障，着力增强服务经济发展的能力。

　　提高财政运行质量。深化财税体制改革，严格落实“营改增”等各项税改政策。坚持依法征税，确保应收尽收、均衡入库，提高税收比重，培植稳固的财源基础，增加可用财力。牢固树立过紧日子思想，完善预算绩效、国库集中支付改革，严格控制一般性支出，大力压缩“三公”经费，一般公用经费实现“零增长”，会议、接待等支出压缩20%以上，新增财力重点投向民生支出。完善国有资产管理体制，加大政府性资产清理力度，解决好改制企业遗留问题，提高国有资产保值增值率。把控制和防范政府性债务风险作为经济工作的重要任务，完善政府重大项目投融资决策、债务风险预警和监管等机制，严格控制政府直接举债、控制融资成本、控制债务规模，切实提高政府性资金使用效益。

　　提高金融服务水平。按照发展普惠金融的要求，全面落实金融服务实体经济的各项政策措施，建立扩大信贷投放激励机制，引导金融机构加大对产业转型升级和中小企业的信贷支持力度，力争全市新增社会融资总量135亿元，贷款增幅超过全国、全省平均水平。加快企业直接融资步伐，力争市城投公司15亿元企业债券获批发行，新增场外市场挂牌交易企业3户以上，1～2户企业在“新三板”上市。加大融资担保力度，全市融资担保余额35亿元。进一步加强金融市场监管，严厉打击各类非法集资行为，有效防范和化解金融风险。支持金融机构发展，确保每万人网点拥有量继续保持全省前列。

**（九）坚持以人为本，全力保障和改善民生**

　　按照“守住底线、突出重点、完善制度、引导舆论”的思路进一步做好民生工作，让发展成果更多更公平惠及全市人民。

　　认真组织实施民生工程。紧紧围绕群众最关心、最紧迫的民生需要，既尽力而为又量力而行，务实安排民生工程项目，在省确定33项民生工程建设任务的基础上，组织实施菜篮子、棚户区和“三小”改造、精品社区建设3项市级民生工程，共投入资金32亿元。强化资金和质量监管，完善长效机制，确保民生工程发挥持久效益，确保全市民生工程工作继续走在全省前列。

　　加强就业和社会保障工作。加快建立经济发展和扩大就业联动机制，多渠道增加就业岗位，确保全年城镇新增就业2万人，下岗失业人员再就业1.2万人，就业困难人员再就业2900人，高校毕业生就业率达95%以上。加大社会保险扩面征缴力度，巩固城乡居民社会养老保险全覆盖成果，进一步完善基本医疗、工伤、失业、生育保险市级统筹制度，推进城镇居民大病保险，做好社会保险关系转移接续工作，确保各项社会保险待遇按时足额支付。完善市内异地就医即时结算网络，实现与省结算平台联网。加快发行加载金融功能社会保障卡。继续提高城乡低保和农村五保补助标准。进一步做好城乡医疗救助工作。完善社会救助和保障标准与物价上涨挂钩联动机制。推进住房公积金扩面征缴，建成安置房3439套、各类保障性住房6624套，公开透明、公正公平做好公租房分配工作。

　　大力发展各项社会事业。组织实施新一轮学前教育三年行动计划，乡镇公办中心园覆盖率达到85%。启动实施教育信息化三年推进计划，加快中心城区教育布局优化调整，进一步提高中小学办学条件和水平。大力发展现代职业教育，推进黄山电大向开放大学转型，支持黄山职业技术学院加强专业建设、扩大办学规模，支持黄山学院提高办学层次。加快医疗基础设施建设，市中医院、市二院完成搬迁，完成市医院综合病房大楼主体工程建设。深入开展爱国卫生运动。抓好重大疾病防控。稳定低生育水平，提高出生人口素质，人口自然增长率控制在5.5‰以内。广泛开展全民健身运动，积极备战省全运会和省残运会。推动军民融合式发展，抓好国防教育、国防动员、后备力量建设和人民防空工作。积极发挥工会、共青团、妇联等人民团体的重要作用。关心老龄工作，支持残疾人事业，完善农村留守儿童、妇女、老人关爱帮扶体系。做好民族、宗教、档案、地方志等工作。

**（十）创新社会治理，切实维护社会和谐稳定**

　　建立健全以民意为导向的社会治理机制，提高社会治理科学化水平，确保人民安居乐业、社会安定有序。

　　积极创新社会治理。深入开展“六五”普法，扎实推进基层民主法制建设，做好第九届村民委员会换届选举工作。进一步完善人民调解、行政调解、司法调解联动的大调解工作体系，建立健全社会稳定风险评估机制。巩固“五级书记带头大走访”活动成果，扎实开展信访积案化解年活动，落实领导干部开门接访、带案下访等制度，全面推广“两个中心”、“两个工作站”建设经验，深入开展矛盾纠纷排查化解，妥善解决人民群众反映强烈的突出问题。深入推进社区矫正和安置帮教工作，抓好社区矫正示范县（区）创建。不断完善社会治安防控体系建设，依法严厉打击各类违法犯罪行为，确保社会大局和谐稳定。

　　强化公共安全体系建设。全面落实安全生产责任制，强化政府监管职责，落实企业主体责任，进一步加强重点行业、重点领域、重点企业的安全监管工作，严格落实建设项目安全生产评估。严厉打击各种非法违法生产经营活动，深入开展中小学校、公众聚集场所、旅游、道路交通、矿山、化工、烟花爆竹等重点领域、行业安全生产专项整治，消除各类隐患，力保12项安全生产指标控制在省下达范围之内。加强网络舆情管理和应对。加大食品药品安全监管力度。强化应急平台和应急专业队伍建设，加强地震、气象、防灾减灾工作，提高应急保障能力。

**三、切实加强政府自身建设**

　　转型发展的新形势，改革创新的新任务，对政府自身建设提出了新的更高要求。我们要扎实开展党的群众路线教育实践活动，切实解决“四风”方面突出问题，着力打造为民务实清廉政府。

**一要强化宗旨意识，树立一心为民的导向。**始终坚持以人为本，把群众路线作为根本工作路线，坚持问计于民，深入群众、深入基层，充分尊重群众首创精神；坚持问政于民，畅通民意表达渠道，着力解决群众反映的突出问题；坚持问需于民，把更多的精力投入到公共服务上，把更多的财力倾斜到改善民生上，多办让人民满意的好事、实事，不断增强人民幸福感和社会和谐度。

**二要强化发展意识，树立真抓实干的导向。**始终坚持以经济建设为中心，把加快发展作为政府工作的第一要务。切实增强忧患意识、问题意识，在全省发展的大格局中对标审视，正视差距和不足，直面困难和问题，增强不进则退、慢进亦退的危机感和紧迫感。切实增强机遇意识、责任意识，充分认识和把握日益凸显的生态优势、文化优势和区位优势，充分认识和把握这些年来“三位一体”建设所打下的良好基础，肩负起加快发展的历史责任，不负人民群众过上美好生活的期待。切实增强改革意识、进取意识，始终保持改革创新的勇气和锐气，始终保持争创一流、迎难而上的干劲和斗志，不断解决前进道路上的困难和问题。按照“一分部署、九分落实”的要求，切实提高政府执行力，对看准的事、定下来的事，说干就干、干就干好。

**三要强化法制意识，树立按章办事的导向。**始终牢记权力意味着责任，在制度面前不擅自用权，在规矩面前不变通处理。政府工作人员要带头学法、守法、用法，严格按照法定的权限和程序行使权力、履行职责。进一步建立健全重大决策调查研究、专家论证、听证公示等制度，完善依法决策、科学决策、民主决策机制，以制度巩固成果，用机制规范行为。坚持重大决策出台前向人大及其常委会报告、与政协民主协商，自觉接受人大及其常委会的法律监督、工作监督和政协的民主监督，办好人大代表议案、建议和政协提案，认真落实人大决议决定和审议意见，注重采纳政协调研成果和意见建议。广泛听取民主党派、工商联、无党派人士和各人民团体的意见，主动接受社会公众和新闻舆论监督。

**四要强化廉洁意识，树立干净干事的导向。**坚持“标本兼治、综合治理、惩防并举、注重预防”的方针，认真落实党风廉政建设责任制，全面开展廉政风险防控，加强对重点领域和关键环节的权力制约，严格执行项目招投标、国有土地出让、产权交易、政府采购和政府性资金的管理制度，把权力关进制度的笼子。严格执行中央“八项规定”和省、市委有关具体要求，认真落实惩防体系2013～2017年工作规划，进一步加强行政监察、审计监督和纠风治乱工作，严肃查处各类违纪违法行为。

　　各位代表！

　　打造美丽中国先行区，既要把黄山建美，更要把黄山变富，这是人民群众之期盼，这是各级政府的职责。让我们在市委的坚强领导下，进一步解放思想，改革创新，为全面完成今年各项目标任务而奋力拼搏，为加快建设现代国际旅游城市、全面建成小康社会而不懈努力！
